# Intractable ascites associated with mycophenolate in a simultaneous kidney-pancreas transplant patient: a case report

**DOI:** 10.1186/s12882-017-0757-5

**Published:** 2017-12-12

**Authors:** Nina T. Weber, Ali Sigaroudi, Alexander Ritter, Andreas Boss, Kuno Lehmann, David Goodman, Stefan Farese, Stefan Weiler, Thomas F. Mueller

**Affiliations:** 1 Clinic of Nephrology, Departments of Medicine, University Hospital Zurich and University of Zurich, Zurich, Switzerland; 2 Department of Clinical Pharmacology and Toxicology, University Hospital Zurich and University of Zurich, Zurich, Switzerland; 3 Radiology, University Hospital Zurich and University of Zurich, Zurich, Switzerland; 4 Surgery, University Hospital Zurich and University of Zurich, Zurich, Switzerland; 5 Department of Nephrology, St Vincent’s Hospital Melbourne, Melbourne, Australia; 6 Department of Nephrology, Burgerspital, Solothurn, Switzerland

**Keywords:** Sodium mycophenolate, Mycophenolic acid, Ascites, Transplantation, Case report

## Abstract

**Background:**

Mycophenolic acid (MPA), either given as an ester pro-drug or as an enteric-coated sodium salt, is the most commonly prescribed anti-proliferative immunosuppressive agent used following organ transplantation and widely applied in immune-mediated diseases. Clinicians are well aware of common adverse reactions related to MPA treatment, in particular diarrhea, leukopenia and infections. Here we report a case of severe, persistent ascites associated with MPA treatment. The otherwise unexplained and intractable ascites, requiring repeated paracenteses for more than 8 months, rapidly ceased with stopping the MPA treatment. To our knowledge this is the first case of severe ascites associated with MPA treatment reported in the scientific literature.

**Case Presentation:**

A 45-year old female with type 1 diabetes mellitus received a simultaneous kidney-pancreas transplant. The surgery was uneventful. However, post-operatively she developed severe transudative ascites requiring in total more than 40 paracenteses treatments draining in the average 2.8 l of ascites fluid. The ascites formation persisted despite exclusion of a surgical complication, fully functioning kidney and pancreas allografts, lack of any significant proteinuria, normalization of circulating albumin levels, intensive use of diuretics and deliberate attempts to increase the intervals between the paracentesis treatments. Various differential diagnoses, including infectious, hepatic, vascular and cardiac causes were ruled out. Nine months after surgery enteric-coated mycophenolate sodium was switched to azathioprine after which ascites completely resolved. When mycophenolate was recommenced abdominal fullness and weight gain reoccurred. The patient had to be switched to long-term azathioprine treatment. More than 1 year post-conversion the patient remains free of ascites.

**Conclusion:**

MPA is the most widely used antimetabolite immunosuppressive agent. We suggest to consider MPA treatment in the differential diagnosis of severe and unexplained ascites in transplant and non-transplant patients.

## Background

Mycophenolic acid (MPA) is the key anti-proliferative immunosuppressant used in organ transplant recipients. MPA is available as an ester pro-drug mycophenolate mofetil (MMF, CellCept®, Roche Pharma, Reinach, CH) or as an enteric-coated sodium salt mycophenolate sodium (EC-MPS, Myfortic®, Novartis Pharma, Rotkreuz, CH). MPA is a reversible inhibitor of inosine monophosphate dehydrogenase (IMPDH). The anti-proliferative effect on lymphocytes is the predominant mechanism by which MPA exerts its immunosuppressive effects [1, 2]. The major adverse effects associated with MPA include gastrointestinal, hematological and infectious complications, with diarrhea and leukopenia being clinically the two most relevant [3, 4]. In this report we describe a case of severe, refractory transudative ascites occurring in a female kidney-pancreas transplant recipient associated with EC-MPS treatment. Ascites resolved after medication discontinuation and likely recurred with medication re-challenge. To our knowledge this the first case describing severe ascites in association with mycophenolate therapy.

## Case Presentation

### Patient information

A 45-year old Caucasian female (weight 53 kg, BMI 19.6 kg/m^2^) with longstanding type 1 diabetes mellitus received a simultaneous kidney-pancreas transplant. The patient had no known drug allergies. She neither smoked nor had a history of heavy alcohol consumption. Prior to transplantation the patient had been on peritoneal dialysis for 5 months without complications. The Tenckhoff catheter was removed during transplantation surgery.

The initial immunosuppressive regimen consisted of anti-thymocyte globulin, steroids, tacrolimus with a target 12-h trough level of 10–15 μg/L, and EC-MPS 720 mg twice daily. There were no peri- or post-operative surgical complications. Dual graft function was immediate and excellent with normalization of estimated glomerular filtration rate to 55–65 ml/min per 1.73m^2^ within 3 days and good glycemic control without exogenous insulin use. Within 2 weeks after transplantation, however, the patient complained of anorexia, nausea and abdominal fullness. Dyspnea was denied.

### Physical exam, diagnostic assessment and timeline

Physical examination revealed shifting dullness consistent with ascites and mild peripheral edema. Abdominal ultrasound confirmed the presence of ascites with normal hepatic parenchyma. There was no portal vein thrombosis and normal hepatopetal portal venous flow. The wedged hepatic venous pressure was 12 mmHg compared to a central venous pressure of 6 mmHg, effectively ruling out portal hypertension or veno-occlusive disease with a pressure gradient of 6 mmHg. Furthermore there were no esophageal or gastric varices seen on upper gastrointestinal endoscopy. Liver stiffness measured by ultrasound-based transient elastography was 6.4 kPa (normal <7 kPa) making fibrosis or cirrhosis very unlikely. Based on the normal findings of flow, morphology, pressures and stiffness a liver biopsy was not performed.

**Fig. 1.**
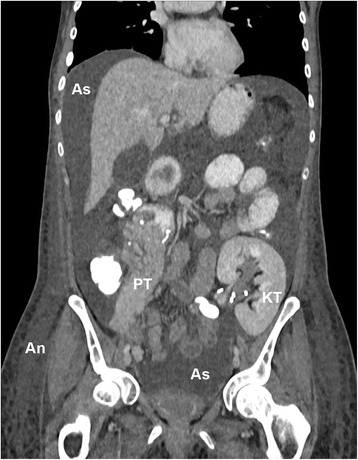
CT-scan in the parenchymal phase after iv-injection of iodinated contrast agent on day 18 after combined kidney-pancreas transplantation. Small bowel is filled with oral contrast-agent, colon structures with rectal contrast agent. The kidney transplant (KT) in the left lower abdomen still exhibits a ureteral stent. In the upper part of the pancreas transplant (PT) the anastomosis to the small bowel is visible. Both, kidney and pancreas grafts display normal contrast agent uptake behavior. Extensive ascites (As) can be seen in the four quadrants of the abdomen, moreover anasarca (An) is shown in the subcutaneous tissue of both flanks

CT scanning of the abdomen was performed that excluded anastomotic leakage from the renal artery and vein or from the duodenal anastomosis to the proximal jejunum. However it confirmed extensive ascites formation in all four abdominal quadrants (Fig. 1). Chest x-rays revealed no signs of pleural effusion. Cardiac ultrasound demonstrated a normal sized left ventricle with a normal ejection fraction of ≥55%. There was mild diastolic dysfunction without features of pulmonary hypertension, right ventricular function and dimensions were normal.

**Fig. 2:**
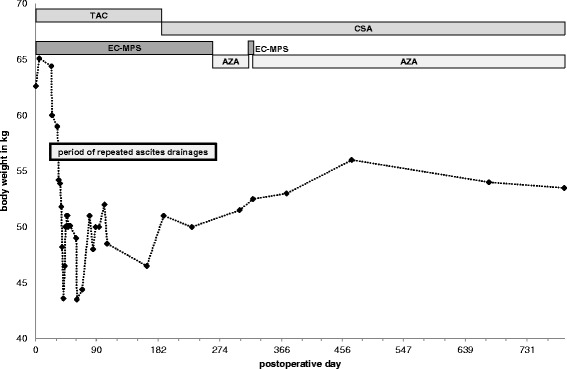
Follow up of body weight (kg, primary axis), postoperative days (x-axis), conversion of treatment from tacrolimus (TAC) to cyclosporine A (CSA), enteric coated mycophenolate mofetil (EC-MPS) to azathioprine (AZA) and time period of repeated paracenteses treatments are shown

Diagnostic and therapeutic paracentesis was performed for the first time 18 days after transplantation and repeated multiple times over the following 8.5 months (Fig. 2). In total more than 40 times ascites fluid was drained first with an indwelling catheter, later by fluid taps, on average 2.8 l each time. The peritoneal fluid was always clear. The cellular counts were low (< 100 cells/*μ*l) with normal distribution and no pathologic cells. Multiple cultures and Gram stains were negative for pathogens. Analysis of ascitic fluid showed a total protein content between 8 and 23 g/L and albumin content between 5 and 17 g/L. The ascitic fluid showed consistently a high serum-to-ascites albumin gradient (SAAG, average of 23 g/l, range between 17 and 28 g/l), indicating portal hypertension.

Repeated measurements of both pancreatic amylase and creatinine in the ascitic fluid were performed to rule out leakage from the pancreatic or ureteric anastomosis, respectively. The amylase concentration was mildly increased on one occasion (ascites amylase 92 U/L, serum amylase 155 U/L, ratio of 0.59). Laboratory tests including INR (international normalized ratio), platelet count, thyroid and liver function tests (ALT and bilirubin) were within the normal ranges. The complete blood counts were normal except for a slight leukopenia attributed to the immunosuppressive therapy. C-reactive protein (CRP) was elevated in the first two post-operative months and normalized later on. Initial urine protein levels were between 1.0 and 1.8 g per day (in spot urine) which quickly resolved to 0.1 to 0.35 g per day over the period of observation. Serological testing for hepatitis A, B and C as well as HIV was negative. MPA 12-h through levels were 1.8 and 3.9 mg/L in two different analyses (using CEDIA® Mycophenolic Acid Assay, Fisher Scientific Inc., Reinach, CH). There is no validated target range but these results do not suggest overdosing.

### Differential diagnosis

After ruling out technical complications, persistent edema and ascites were explained by a combination of early post-operative factors associated with simultaneous kidney-pancreas transplant surgery in a small-sized patient, such as high volume state, low oncotic pressures, activation of the inflammation cascade and previous peritoneal dialysis treatment. However, despite significant volume loss, increasing serum protein levels, full clinical recovery, and periods of deliberate withholding paracentesis despite abdominal distension and distress to prevent a fluid circle of removal and refilling, the ascites formation and need paracentesis persisted. The high SAAG levels suggested portal hypertension-related ascites but both hepatic and cardiogenic causes were extremely unlikely given the normal findings on imaging and pressure measurements. Infectious or malignant causes were ruled out as far as possible. Given the fact that the patient performed peritoneal dialysis with high transporter status only for a short period of time pre-transplant makes peritoneal sclerosis as contributing factors to ascites formation extremely unlikely [5]. The literature suggests nephrogenic ascites in the setting of kidney transplantation mainly in association with rejection and unrecognized heart dysfunction, neither of which was present in our case [6, 7]. One report from 2012 discusses a very rare case of nephrogenic ascites due to an exophytic spongiform lesion on the surface of the renal allograft causing massive transudate ascites formation [8]. The patient was treated by cauterization of the majority of the surface of the allograft, which lead to an involution of the lesions and finally resolution of ascites formation.

All those potential causative factors, as discussed in a recent review of post-transplant ascites [6], were seen as unlikely explanations for the persistence and severity of ascites in our patient.

### Interventions

Paracentesis was necessary every 1–4 weeks for several months tapping up to 8 l of fluid each time. A trial with diuretic treatment with spironolactone did not achieve a sustained effect. In addition deliberately trying to prolong the intervals between paracenteses treatments were not tolerable due to significant weight gain and discomfort for the patient. Overall the patient became progressively more distressed with abdominal distension and the need for repeated paracentesis. Based on the Swiss drug information registry indicating an association of tacrolimus with the development of ascites and pleural effusions the immunosuppressive regimen was changed 6 months after transplantation by substituting cyclosporine A for tacrolimus. This did not reduce the ascites or the need for paracentesis. Further literature review noted the association of EC-MPS with abdominal tension in <1/100 to >1/1000 cases (Swiss product information). Therefore, despite the increased immunological risk and potential serious consequences, EC-MPS was switched to azathioprine 8.5 months after transplantation (see Fig. 2). This conversion was associated with a rapid resolution of the ascites. Seven days after EC-MPS was stopped the last paracentesis was performed. While on azathioprine the patient became progressively neutropenic despite reducing the daily dose and normal thiopurine methyltransferase enzyme activity. Finally azathioprine was discontinued after 1.5 months of therapy and EC-MPS 360 mg twice daily was recommenced. This was associated with the immediate sensation of abdominal tension and weight gain. Withdrawal of EC-MPS again led to rapid improvement of the symptoms. No other medications were changed during this time period implicating re-exposure to EC-MPS as the likely cause of ascites.

### Follow-up and outcome

This case report suggests a rare adverse reaction of MPA treatment. Our patient developed severe, refractory ascites shortly after transplantation. Despite multiple investigations we could not explain the development and persistence of this intractable ascites extending throughout all four abdominal quadrants. There were no surgical complications, no visible peritoneal or intestinal abnormalities, no abdominal infection, no portal hypertension, and no evidence of impaired hepatic or cardiac function. Switching tacrolimus to cyclosporine did not reduce the ascites. However, the conversion from EC-MPS to azathioprine was associated with the resolution of the ascites. The re-exposure to EC-MPS 6 weeks later immediately caused abdominal tension and weight gain. Therefore, a clear temporal relationship between EC-MPS administration and ascites formation, with cessation of ascites following drug withdrawal and rapid recurrence following re-challenge, was seen. Now, with a follow up of more than 2 years the patient remains free of ascites, her transplant functions are stable, 2 non-complement binding donor-specific antibodies are unchanged and the most recent kidney transplant biopsy shows no significant pathology.

An adverse drug reaction was considered highly probable, categorized as medically important and reported to regulatory authorities. Based on the WHO/CIOMS (World Health Organisation/Council for International Organizations of Medical Sciences) causality scale in Pharmacovigilance the causality between ascites formation and EC-MPS was categorized as “certain” (temporal relationship, positive de- and re-challenge, exclusion of other differential diagnoses).

## Discussion and conclusions

The diagnosis of MPA-induced ascites was based on the results of investigations which excluded alternative causes, the timing of onset and resolution of ascites after discontinuation of therapy and finally the recurrence of symptoms and signs with re-challenge with EC-MPS. Taken together these observations strongly support causality to the suspected adverse drug reaction [9]. MPA, as main constituent of EC-MPS and MMF, defines the largely similar side-effect profile of both drugs [4]. A search in VigiBase, the World Health Organization global database of Individual Case Safety Reports identified from a total of 19,338 reports involving MPA 90 cases of ascites between the years 2000 and 2015. In 88 cases MPA was reported as a suspected drug. In 4 cases “ascites” was the only reported side-effect and in just 2 patients MPA was reported as the only suspected drug. Causality assessment or de- and re-challenge were not reported in the WHO database. To our knowledge there are no cases of severe ascites associated with MPA treatment reported in the scientific literature.

In the presented case we describe MPA-associated ascites formation with positive de-challenge after exclusion of various differential diagnoses. Upon re-challenge with MPA although the patient’s symptoms were highly suggestive of repeated ascites formation we were unable to obtain confirmation of ascites formation prior to drug cessation which was done elsewhere. The impact of MPA re-challenge is therefore not objectively proven, however the rapid resolution of ascites upon initial cessation of MPA strongly supports a causative association. The pathomechanism of the MPA-associated ascites formation in our patients is not clear. Gastrointestinal adverse effects are a common complication of MPA treatment [4]. Known MPA-associated morphological gastrointestinal toxicity includes duodenal villous atrophy in renal transplant recipients [10], mucosal damage of the stomach and erosive or ulcerative enterocolitis [11]. Genetic polymorphisms of MPA metabolism and transport might account for individual differences in side effects. However, clinically severe ascites formation has not been published as a complication of MPA treatment. An increase in capillary permeability was considered as explanation. In rats treated with MMF elevated levels of nitric oxide were detected in the serum as well as in the duodenum. Histopathologic examinations showed villous atrophy and inflammatory cell infiltration. Gastrointestinal disorders among these animals were therefore attributed to local inflammatory reactions which were possibly caused by elevated NO and myeloperoxidase levels [12]. However MPA is better known for its inhibitory effects on inflammation [1, 4]. In a mouse model of pleurisy, MMF suppressed protein levels of inflammatory cytokines such as TNFα, IL1β, VEGFα and IL-17 [4, 13]. In a model of encapsulating peritoneal sclerosis MMF reduced inflammation and neovascularization by inhibiting VEGF and TGFβ1 [14]. Hence, this inhibition of vascular growth factors and inflammatory cytokines would rather lead to a decrease of vascularity, tightening of the blood-peritoneal-barrier, less vasodilatation, reduced capillary leakage and therefore less ascites formation. An inflammation-induced increase in capillary permeability is also not compatible with the high SAAG gradient, low protein content and lack of inflammatory cells in the transudative ascitic fluid in our patient.

In summary MPA treatment should be added to the differential diagnosis of severe ascites. Given the worldwide use of MPA as antimetabolite of choice in immunosuppressive regimens our observation might raise awareness in cases of unexplained ascites formation in transplant as well as non-transplant patients.

### Patient perspective

“Shortly after the surgery when the ascites occurred I felt desperate and unsure about ever leaving the hospital again. It took many weeks until I was finally able to go home but still needed weekly paracenteses treatments as outpatient. During this time I decided to insist with my doctors to resolve this problem because I felt my life had become unbearable. I started studying the drug information sheets of my medication and read about the potential side effect of ascites. I also had an aunt who was a kidney transplant recipient and was treated with cyclosporine A and azathioprine for many years and after switching her to tacrolimus and MPA later in life she felt increased abdominal fullness and volume. With this personal experience and reading about the side effects I suggested to my doctors repeatedly a switch from tacrolimus to cyclosporine A. After this change I felt better in general but the ascites persisted. When the change of MPA to azathioprine was done I slowly felt a relieve of the abdominal tension. The re-challenge with MPA made me then feel “like a balloon” after just a couple of doses.”

“I have never regretted the decision to have a kidney and pancreas transplantation, despite the complicated course I would always do it again. The full glycemic control and cessation of dialysis are important assets in my life that I would not want to miss any more.”

## Abbreviations

ALT: Alanine aminotransferase
CIOMS: Council for International Organizations of Medical Sciences
CRP: C-reactive protein
EC-MPS: Enteric-coated mycophenolate sodium
HIV: Human immunodeficiency virus
IL1-β: Interleukin 1 β
IMPDH: Inosine monophosphate dehydrogenase
INR: International normalized ratio
MMF: Mycophenolate mofetil
MPA: Mycophenolic acid
SAAG: Serum-to-ascites albumin gradient
TNF-α: Tumor necrosis factor α
